# Hydrogen Sulfide Inhibits Bronchial Epithelial Cell Epithelial Mesenchymal Transition Through Regulating Endoplasm Reticulum Stress

**DOI:** 10.3389/fmolb.2022.828766

**Published:** 2022-04-12

**Authors:** Fan Lin, Chengcheng Liao, Jinsheng Zhang, Yun Sun, Weiwei Lu, Yu Bai, Yixuan Liao, Minxia Li, Yongfen Qi, Yahong Chen

**Affiliations:** ^1^ Department of Respiratory and Critical Care Medicine, Peking University Third Hospital, Beijing, China; ^2^ Geriatric Medicine Center, Department of Pulmonary and Critical Care Medicine, Zhejiang Provincial People’s Hospital (Affiliated People’s Hospital, Hangzhou Medical College), Hangzhou, China; ^3^ Key Laboratory of Molecular Cardiovascular Science, Ministry of Education, Peking University Health Science Center, Beijing, China

**Keywords:** hydrogen sulfide, chronic obstructive pulmonary disease, epithelial mesenchymal transition, bronchial epithelial cell, endoplasmic reticulum stress

## Abstract

Epithelial mesenchymal transition (EMT) is a contributing factor in remodeling events of chronic obstructive pulmonary disease (COPD). Hydrogen sulfide (H_2_S) has been implicated in the pathogenesis of COPD, but the effect of H_2_S in regulating EMT and the underlying mechanisms is not clear. In this study, we assessed endoplasmic reticulum (ER) stress markers, EMT markers and associated signal molecules in rat lungs, bronchial epithelial cells, and human peripheral lung tissues to investigate the effect of H_2_S in regulating EMT and the underlying mechanisms. We found that EMT and ER stress occurred in lung epithelial cells, especially in the bronchial epithelial cells of smokers and COPD patients. In cigarette smoke (CS)-exposed rats, intraperitoneal injection of NaHS significantly alleviated CS-induced lung tissue damage, small airway fibrosis, ER stress, and EMT, while intraperitoneal injection of propargylglycine (cystathionine-gamma-lyase inhibitor) aggravated these effects induced by CS. In the nicotine-exposed 16HBE cells, an appropriate concentration of H_2_S donor not only inhibited nicotine-induced ER stress, but also inhibited nicotine-induced enhancement of cell migration ability and EMT. ER stress nonspecific inhibitors taurine and 4-phenyl butyric acid also inhibited nicotine-induced enhancement of cell migration ability and EMT. Both H2S and inositol-requiring enzyme 1 (IRE1) activation inhibitor 4μ8C inhibited nicotine-induced activation of IRE1, Smad2/3 and EMT. These results suggest that H_2_S inhibits CS- or nicotine-induced ER stress and EMT in bronchial epithelial cells and alleviates CS-induced lung tissue damage and small airway fibrosis. The IRE1 signal pathway and Smad2/3 may be responsible for the inhibitory effect of H_2_S.

## Introduction

Chronic obstructive pulmonary disease (COPD) has the characteristics of persistent airflow limitation and respiratory symptoms that result from exposure to noxious gases or particles. Among COPD patients, the small airways have the characteristics of epithelial changes, thickening of the small airway wall, inflammatory and mucous exudates induced airway obstruction, inflammatory cell infiltration into the airway wall, proliferation of airway smooth muscle, and progressive peribronchiolar fibrosis (Van den Berge et al., 2011). Small airway dysfunction is an early feature of lung disease and preceded both the detection of emphysema by imaging methods and spirometric evidence of COPD (Hogg et al., 2004). Moreover, there is convincing pathological evidence that a large number of small airways have been lost before the pathological changes of emphysema (Stockley et al., 2017). Small airway remodeling leads to irreversible small airway dysfunction, participating in the pathogenesis of COPD. Cigarette smoke (CS) induced epithelial-mesenchymal transition (EMT) is one of the mechanisms involved in small airway remodeling (Nowrin et al., 2014). In addition, more *in vivo* (Sohal et al., 2010; Sohal et al., 2011) and *in vitro* (Liu et al., 2010; Veljkovic et al., 2011; Eurlings et al., 2014) studies showed that CS-induced EMT in lung epithelial cells contributed to tissue remodeling in patients with COPD. There is convincing evidence that endoplasmic reticulum (ER) stress can activate classic Smad, Wnt/β-catenin and Src protein kinase families, thus inducing EMT in alveolar epithelial cells (Tanjore et al., 2011; Zhong et al., 2011; Zhang et al., 2012). In ER, glucose-regulated protein: 78 (GRP78) binds to three transmembrane sensor protein inositol requiring enzyme 1 (IRE1), activating transcription factor-6 (ATF6), and PKR-like ER kinase (PERK), maintaining each in its inactive state (Osorio et al., 2013; Lindholm et al., 2017; Chadwick and Lajoie, 2019). During ER stress, GRP78 is released from IRE1, ATF6 and PERK, so these three transmembrane sensor proteins can assume their activated state (Lin et al., 2008; Tanjore et al., 2012; Marciniak, 2017). P-IRE1 splices the mRNA of x-box binding protein 1 (XBP1) to the mature form sec-XBP1, which can activate a series of genes involved in ER-associated protein degradation or protein folding, thus playing a protective role in ER stress. However, the downstream effects of the phosphorylation of IRE1 include activation of c-Jun N-terminal kinase (JNK), which mediates some of the harmful effects such as proliferation, differentiation, carcinogenesis, or apoptosis (Tabas and Ron, 2011; You et al., 2013). Recent studies also reported that CS induced-ER stress plays a very important role in the occurrence and development of COPD (Alam et al., 2014; Geraghty et al., 2016). This notion was confirmed by findings that ER stress markers significantly correlated with lung function in COPD patients (Min et al., 2011). Nicotine, as an important component of cigarette smoke extract, is directly associated with COPD. Evaluation of electronic nicotine delivery systems in different models has demonstrated involvement in pathways related to chronic pulmonary diseases (Canistro et al., 2017; Singh et al., 2019; Mikheev et al., 2020). Inhaled nicotine induces bronchial epithelial cell apoptosis and senescence *via* reactive oxygen species mediated autophagy impairment in COPD (Bodas et al., 2016). Nicotine can promote EMT in lungs. Maternal nicotine exposure induces EMT in rat offspring lungs (Chen et al., 2015). Nicotine can increase malignancy through EMT in lung cancer (Dasgupta et al., 2009; Pillai et al., 2015; Zhang et al., 2016; Du et al., 2018). Nicotine can directly induce ER stress response (Pelissier-Rota et al., 2015; Barra et al., 2017; Gonzales et al., 2021; Jiang et al., 2021). In our previous study, we confirmed that nicotine concentration and time dependently increased the expression of ER stress associated apoptosis marker in human bronchial epithelial cells (Lin et al., 2017).

Hydrogen sulfide (H_2_S), known for its poisoning effect, is now recognized as an endogenous gaseous transmitter in health and disease (Wang, 2002). H_2_S is involved in regulating the tension in airway smooth muscle and has anti-oxidation, anti-inflammation, and anti-apoptosis effects in COPD (Suzuki et al., 2021). According to our previous research, exogenous administration of H_2_S protected against CS-induced bronchial epithelial cell apoptosis through inhibiting ER stress (Lin et al., 2017). H_2_S may play a protective role in bronchial epithelial cells through regulating ER stress. Recent research reported that H_2_S can inhibit EMT and oxidative stress, thus preventing the airway remodeling induced by CS in mouse lungs. H_2_S can also attenuate CS-induced EMT by inhibiting the activation of the transforming growth factor β1 (TGF-β1) -Smad3 signaling pathway (Guan et al., 2020). Furthermore, Fang LP found that exogenous administration of H_2_S suppressed TGF-β1 mediated EMT and preincubation with H_2_S decreased the phosphorylation of Smad2/3 induced by TGF-β1 in human lung carcinoma (A549) cells (Fang et al., 2010). Our previous research found that exogenous H_2_S could also inhibit TGF-β1 induced human bronchial epithelial cell morphological changes and EMT (Liao et al., 2015). All these results suggest that exogenous administration of H_2_S can inhibit EMT in the lung. H_2_S is mainly produced by cystathionine-gamma-lyase (CSE) in respiratory organs. Our group has proposed for the first time that CSE expression is decreased in the lungs of smokers and COPD patients compared with nonsmokers (Sun et al., 2015). The effect of endogenous H_2_S in the lung and its role in the pathogenesis of EMT and airway remodeling induced by CS remains unclear.

We therefore hypothesized that endogenous H_2_S might inhibit CS-induced EMT in the lung and that H_2_S might regulate ER stress to suppress bronchial epithelial cell EMT, which plays an essential role in the small airway fibrosis of COPD. Therefore, we assessed the ER stress and EMT markers in human lung tissues, rat lung tissues from a COPD rat model established by passive CS exposure, and 16HBE cells exposed to nicotine in order to investigate whether H_2_S can inhibit EMT of bronchial epithelial cells by regulating ER stress and possible signal pathways.

## Materials and Methods

### Patients

Human peripheral lung tissue samples from 21 patients who underwent thoracic surgery at Peking University Third Hospital from April 2012 to July 2014 were included in this research. The protocol was authorized by Peking University Third Hospital Ethics Committee (IRB00006761-2012029). All participants were fully informed of the purpose and duration of the research and provided written informed consent. The diagnosis of COPD was made according to the GOLD (Global Initiative for Chronic Obstructive Lung Disease) criteria (GOLD, 2009). Subject age, smoking index, height, weight, and the pulmonary function indexes of forced expiratory volume in 1 s (FEV1), forced vital capacity (FVC) % predicted, and FEV1/FVC were listed in Supplementary Table S1 to describe the clinical features of patients. All the patients were clinically stable for 4 weeks without acute pulmonary infection, had not received any chemotherapy before the study, and did not have metastasis, obstructive atelectasis, other pulmonary diseases, or severe diseases in other systems. All of the human lung tissues were obtained at least 5 cm away from the tumor margin. The pathological examination confirmed that these samples presented lung structure without inflammation or metastasis.

### Animal Model

All animal care and experimental protocols were in compliance with the ethical procedures and policies approved by the Animal Care and Use Committee of the National Research Center and the Third Hospital, Peking University Guide for the Care and Use of Laboratory Animals.

Adult male Sprague–Dawley rats were supplied by the Animal Center, Peking University Health Science Center. All rats were housed in groups of 4 to a cage with sufficient oxygen, at a room temperature of 18–25°C and a room humidity of 35–50%. A total of 32 adult male Sprague–Dawley rats, weighing about 200–250 g, were randomly allocated into four groups (each *n* = 8) for treatment: control, CS, propargylglycine (PPG) + CS and NaHS + CS. Except in the control group, the rats in other groups were exposed whole-body to CS generated by 20 commercial unfiltered cigarettes daily. Exposure time was 4 h a day for 7 days a week in the 4 months. The CS group rats intraperitoneally injected with PPG (CSE inhibitor) was considered to the PPG + CS group. Freshly prepared PPG (37.5 mg/kg body weight/day) was intraperitoneally administered 30 min before CS-exposure in the PPG + CS group since the beginning of the third month. The CS group rats intraperitoneally injected with NaHS (H_2_S donor) was considered part of the PPG + CS group. Freshly prepared NaHS (14 μmol/kg body weight/day) was intraperitoneally administered 30 min before CS-exposure in the NaHS + CS group since the beginning of the third month. The control and CS groups comprised rats with an intraperitoneal injection of saline.

### Cell Culture and Treatment

The 16HBE14o- (16HBE) human bronchial epithelial cell line was purchased from Shanghai Bogoo Biotechnology Co., Ltd. (China). 16HBE cells were maintained in a complete RPMI1640 growth medium supplemented with 10% fetal bovine serum (Gibco, Waltham, MA, United States), 100 mg/ml streptomycin (Gibco, Waltham, MA, United States), 100 U/ml penicillin (Gibco, Waltham, MA, United States), and 2 mM l-glutamine (Gibco, Waltham, MA, United States) in a humidified atmosphere with 5% CO_2_ at 37°C. Cells were starved in serum-free medium for 24 h before drug treatment. Taurine (10 mM), 4-phenyl butyric acid (4PBA) (5 mM), morpholin-4-ium-4-methoxyphenyl-(morpholino)-phosphinodithioate (GYY4137), and NaHS were dissolved in phosphate buffer solution, nicotine, and 4μ8C were prepared in dimethyl sulfoxide (DMSO).

### Immunohistochemistry

Lung tissue specimens were fixed in formalin, embedded in paraffin, then cut into 4–6 μm sections and stained with haematoxylin-eosin. The sections were incubated with primary antibodies anti-E-cadherin (1:200), anti-GRP78 (1:200), anti-alpha-smooth muscle actin (alpha-SMA) (1:50), anti-p-IRE1 (1:50) or anti-Vimentin (1:50) at 4°C for 24 h. Anti-goat IgG-conjugated with DAB was used as the secondary antibody at a dilution of 1:100. Pre-immune IgG isotope served as a negative control.

### Western Blot Analysis

Protein samples prepared from lung tissue samples and the human bronchial epithelial cell line 16HBE were resolved by SDS-PAGE (10% acrylamide gel) and then transferred to a nitrocellulose membrane. The nitrocellulose membrane was then incubated with the primary antibodies anti-CSE (1:2,000), anti-zonula occludens-1 (ZO-1) (1:1,000), anti-E-cadherin (1:2,000), anti-alpha-SMA (1:500), anti-p-IRE1 (1:1,000), anti-IRE1 (1:1,000), anti-GRP78 (1:3,000), anti-sec-XBP1 (1:500), anti-ATF6 (1:500), anti-p-Smad2/3 (1:500), anti-Smad2/3 (1:500), anti-p-JNK (1:500), anti-JNK (1:500), anti-β-actin (1:3,000) or anti-glyceraldehyde-3-phosphate dehydrogenase (GAPDH) (1:5,000) overnight, then secondary antibody for 1 h. The enhanced chemiluminescence was applied to visualize the reaction. Expression levels of proteins were normalized to those of GAPDH or β-actin.

### Hematoxylin and Eosin and Picrosirius Red Staining

The histopathological changes of lung tissue were measured by Hematoxylin and Eosin (HE) staining. The pathological scores of small airways were measured by separate evaluation of eight variables according to Cosio M (Cosio et al., 1978). The airways of 2 mm or less in diameter were measured by the separate evaluation of eight variables, including the degree of cell and mucus-induced airway lumen occlusion, which was corrected for lung inflation and expressed as a percent of occluded airway lumen. The presence or absence of mucosal ulcers in small airways was observed and recorded for each airway. The data is shown as a percentage of airways with mucosal ulcers. The remaining variables included squamous-cell metaplasia and goblet-cell metaplasia of the epithelium and changes in the airway wall, which included the amount of connective tissue, muscle, and pigment, and the degree of inflammatory-cell infiltration. Each of these changes was assigned a score ranging from 0 to 3, and these scores were summed to achieve an overall score. Then we expressed the sum as a percent of the maximum possible scores. The final pathological score was assigned by the simple addition of a score for each of the eight variables in each case.

The collagen deposition in small airways was measured by Picrosirius red staining. The prepared sections were photographed under transmitted or polarized light on a Leica DM IRB fluorescence microscope. Color photographic slides made with tungsten film were converted to digital images by scanning (Wang et al., 2000). By using this technique, loosely packed collagen fibers appear green, whereas tightly packed collagen fibers appear yellow-red and less mature. All these images are then analyzed by Image Pro-plus 6.0 image analysis software. This procedure was applied to a total of five fields per sample on a minimum of four animals per group. All these data were compiled for statistical analyses (Wang et al., 2000). The ratio of collagen area to the whole lung tissue area in each image reflected the collagen content.

### Wound Healing Assay

The migration ability of human bronchial epithelial cells was measured by a wound healing assay. 16 HBE cells were plated on 60 mm Petri dishes and grew to a confluent monolayer. A single layer of cells was scraped in a straight line with the tip of a sterile pipette (1 ml) on every Petri dish. Then the dishes were washed twice with PBS and replaced with 5 ml of medium containing different drugs. 16 HBE cells were pretreated with NaHS (200 µM) for 0.5 h and then stimulated with nicotine in the Nicotine + NaHS group, and 16 HBE cells were pretreated with taurine (10 mM) for 0.5 h and then stimulated with nicotine in the Nicotine + Taurine group. All the dishes were placed at 37°C for 12 h (Liang et al., 2007). The images were photographed using an inversion fluorescence microscope. Six independent experiments were conducted. The largest migration distance and the closure rate were calculated by IPP software after the wound healing assay. The percentage of the wound healing was calculated as the largest migration distance (the shortest width of the wound at 0 h—the shortest width of the wound at 12 h/the width of the wound at 0 h) and the closure rate (the area of the wound at 0 h—the area of the wound at 12 h/the area of the wound at 0 h).

### Statistical Analysis

The data is expressed as mean ± SD (for normally distributed data) or median (for non-normally distributed data). For normally distributed data, comparisons among more than 2 groups were analyzed by one-way analysis of variance followed by the Student-Newman-Keuls test. For non-normally distributed data, the Wilcoxon signed rank test was used. *p* < 0.05 was considered statistically significant.

## Results

### The Expression and Localization of Epithelial Mesenchymal Transition Markers and Endoplasmic Reticulum Stress Markers in Human Lung Tissues

Human peripheral lung tissues were obtained from 21 patients, including 7 smokers with COPD, 7 non-COPD smokers who had normal lung function, and 7 non-smokers who had never smoked. The average age of participants was 65.62 years old. There was no difference in age and weight among the three groups. The smoking index did not differ between non-COPD smokers and smokers with COPD. Forced expiratory volume in the first second/forced vital capacity (FEV_1_/FVC) and FEV_1_% were significantly lower in smokers with COPD compared with non-COPD smokers and non-smokers (Supplementary Table S1).

**FIGURE 1:**
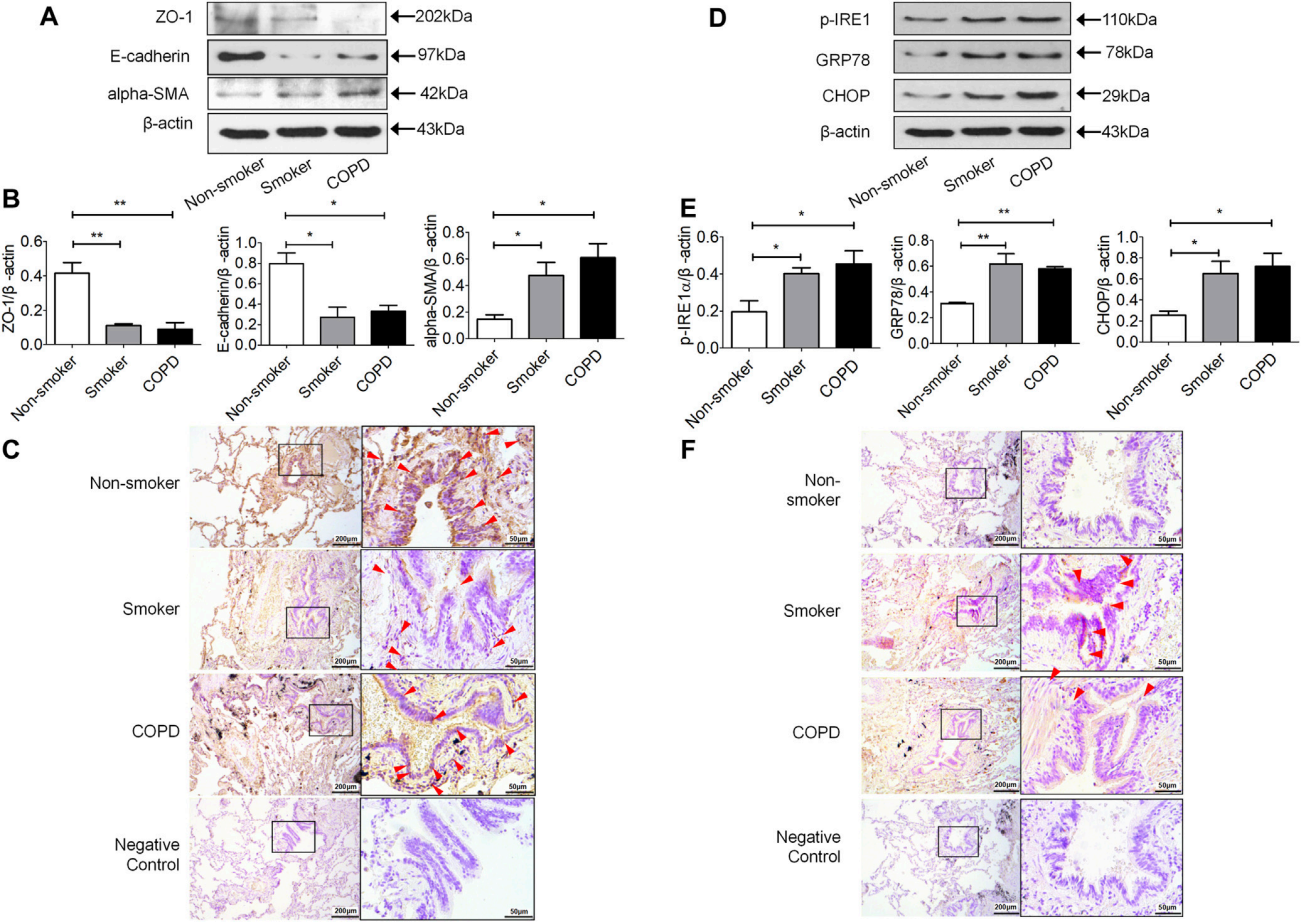
The expression of mesenchymal differentiation markers increased in the lung tissue of smokers and COPD patients. **(A,B)** Western blotting analysis of ZO-1, E-cadherin and alpha-SMA protein expression in the lung tissue of nonsmokers, smokers, and COPD patients, and relative intensity normalized to the expression of β-actin (*n* = 3, respectively, in each group). The alues are expressed as mean ± SEM. *p < 0.05, **p < 0.01 vs. non-smoker group. **(C)** Immunolocalization of E-cadherin in human lung tissues. E-cadherin (red arrow) was localized to the cytoplasm of lung epithelial cells with lighter staining in lung tissue of smokers and COPD patients as compared to non-smokers. Representative images (*n* = 4, respectively, in each group). Original magnification ×100 and ×400. The expression of ER stress markers increased in the lung tissue of smokers and COPD patients. **(D,E)** Western blotting analysis of p-IRE1, GRP78 and CHOP protein expression in lung tissue of nonsmokers, smokers, and COPD patients, and relative intensity normalized to the expression of β-actin (*n* = 3, respectively, in each group). Values are expressed as mean ± SEM. *p < 0.05, **p < 0.01 vs. non-smoker group. **(F)** Immunolocalization of p-IRE1 in human lung tissues. P-IRE1 (red arrow) was expressed in lung epithelial cells and smooth muscle cells, and p-IRE1 staining was significantly more intense and frequent in the lung tissue of smokers and COPD patients as compared to non-smokers. Representative images (*n* = 4, respectively, in each group). Original magnification ×100 and ×400.

The epithelial cell markers E-cadherin and ZO-1 were decreased and the mesenchymal phenotypic marker alpha-SMA was increased in the lung tissues of smokers and COPD patients compared to non-smokers (Figures 1A,B). Immunohistochemistry showed that E-cadherin was located in bronchial and alveolar epithelial cells. It was strongly stained in non-smokers and decreased in non-COPD smokers and smokers with COPD (Figure 1C). P-IRE1 and GRP78 were increased in the lung tissue of non-COPD smokers and smokers with COPD compared with non-smokers (Figures 1D,E). Immunohistochemistry showed that p-IRE1 was expressed in lung epithelial cells and smooth muscle cells. It was stronger in stained non-COPD smokers and smokers with COPD compared with non-smokers (Figure 1F).

### H_2_S Alleviated Chronic Cigarette Smoke Exposure Induced Lung Pathological Damage and Small Airway Fibrosis

A western blot showed the protein level of CSE significantly decreased in the lung tissue of the PPG + CS group compared with the other three groups. There was no significant difference in CSE protein expression between control, CS, and NaHS + CS groups (Supplementary Figure S1).

Our previous study found that the NaHS + CS and NaHS alone groups showed increased plasma H_2_S levels than the control group. Whereas, the PPG + CS group showed a decreased plasma level of H_2_S compared to the CS-alone group. The CS group showed higher CSE protein expression in lung tissue and plasma H_2_S levels as compared to the control group. However, there was no significant difference in the level of H_2_S in rat lung tissue in groups (Chen et al., 2011).

**FIGURE 2:**
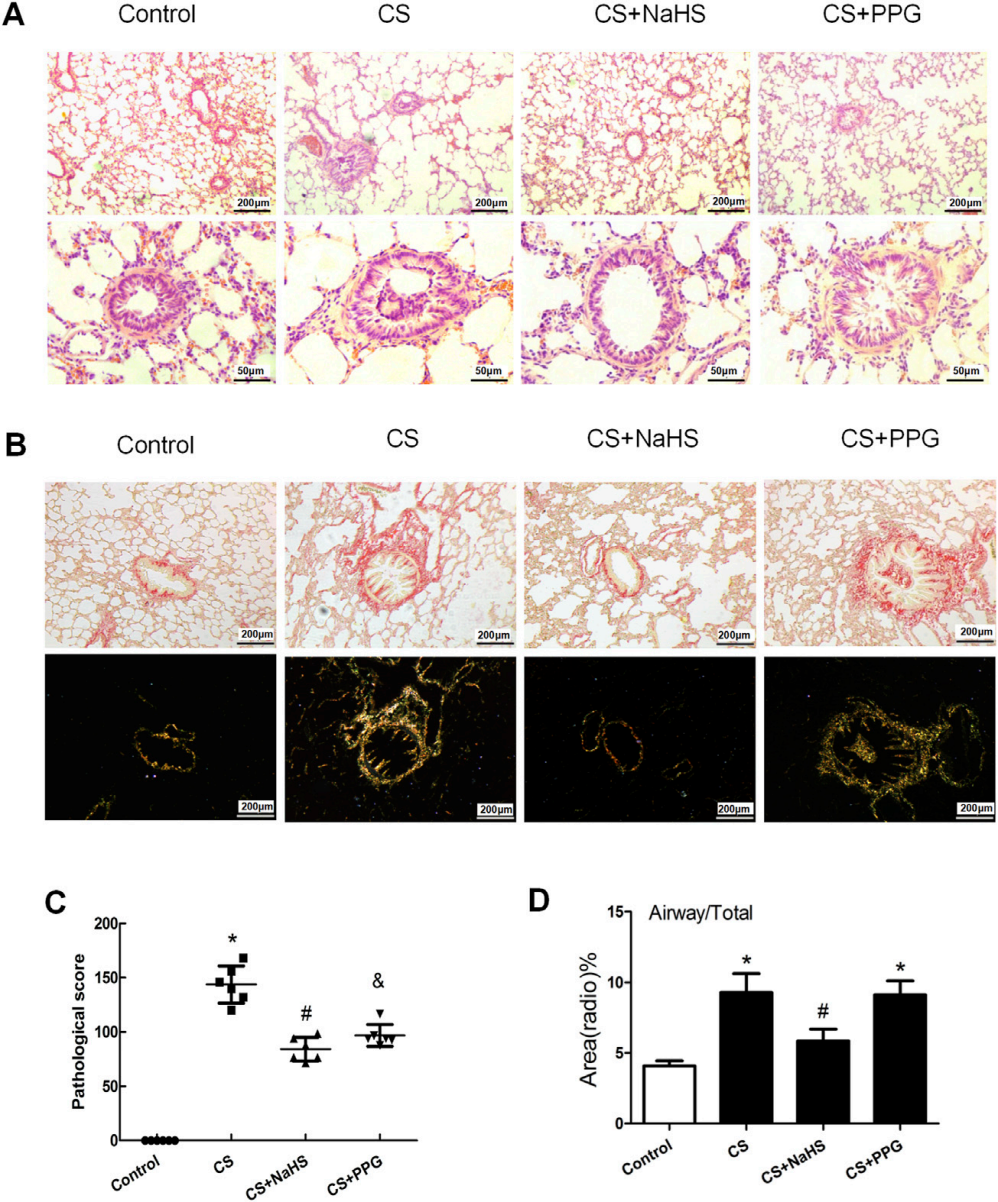
Changes in lung pathology and small airway fibrosis in rats. **(A)** Lung tissue sections were stained with hematoxylin and eosin and examined under light microscopy. Original magnification ×100 and ×400. **(B)** Lung tissue sections were stained with Picrosirius red staining and examined on bright field microscopy (above panels) and polarization microscopy (down panels). Original magnification ×100. **(C)** Airway obstruction, necrotic epithelium, goblet metaplasia, inflammatory cell infiltration, and collagen deposition were seen in the CS group. Emphysema, such as thin or faulted alveolar walls, was seen in the COPD group. Compared with the control group, the pathological score of the small airway was significantly elevated by 42.7% in the CS group (*p* < 0.01), but decreased by 17.9% after NaHS intervention (*p* < 0.05). Results are presented for *n* = 8 mice per group and of 3 independent experiments (*n* = 3, respectively, in each group). *p < 0.05, CS group and PPG + CS group vs. Control group. ^#^ *p* < 0.05, NaHS + CS group vs. CS group. ^&^ *p* < 0.05, PPG + CS group vs. CS group. **(D)** Bar graphs summarizing quantification of small airway fibrosis depended on the Picrosirius red staining and examined on polarization microscopy. Compared with the control group, the collagen deposition around the airway wall was significantly increased in the CS group and the PPG + CS group. The NaHS + CS group had less collagen deposition compared with the CS group. *p < 0.05 vs. Control group and ^#^ *p* < 0.05 vs. CS group.

HE staining and the pathological scores of small airways showed that compared with the control group, the lung tissue of passive smoking rats showed pathological damage. Compared with CS alone, the PPG + CS group showed more severe pathological damage to lung tissue, and the NaHS + CS group showed milder pathological damage to lung tissue (Figures 2A,B). The degree of small airway fibrosis (collagen I and III) as determined by the Piscosirius-red polarization method was significantly higher in the COPD rat model established by passive smoking exposure. Intraperitoneal injection of NaHS in the NaHS + CS group significantly alleviated small airway fibrosis compared with CS alone, while intraperitoneal injection of PPG in the PPG + CS group exacerbated small airway fibrosis compared with CS alone (Figures 2C,D).

### Endogenous H_2_S Inhibited CS-Induced Lung Epithelial Cell Epithelial Mesenchymal Transition in Rats

**FIGURE 3:**
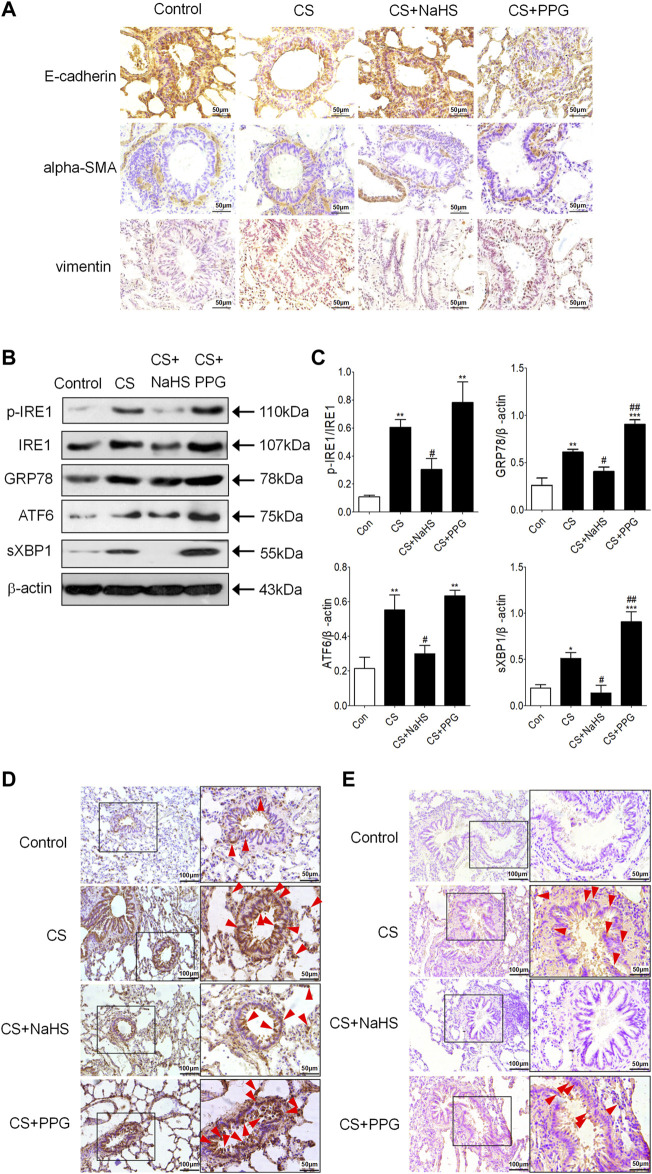
Endogenous H_2_S protects against EMT and ER stress in the lung tissues of CS-exposed rats. **(A)** E-cadherin is localized to the respiratory epithelial cell. Compared with the control group, E-cadherin was lighter in the CS group and the PPG + CS group. The NaHS + CS group had more intense E-cadherin compared with the CS group. Alpha-SMA is localized in the cytoplasm of mesenchymal cells, and Vimentin is localized in the cytoplasm of mesenchymal cells and epithelial cells. Compared with the control group, alpha-SMA and Vimentin were more intense in the CS group and the PPG + CS group, especially in bronchial epithelial cells. The NaHS + CS group had lighter alpha-SMA and Vimentin compared with the CS group. **(B,C)** Western blotting analysis of ER stresses markers p-IRE1, GRP78, ATF6 and sec-XBP1 protein expression in the lung homogenates of rats. Results are presented of 3 independent experiments (*n* = 3). Values are expressed as mean ± SEM. *p < 0.05, **p < 0.01 vs. control group and ^**#**^ *p* < 0.05 vs. CS group. **(D)** GRP78 (red arrow) is localized in the cytoplasm of respiratory epithelial cells and mesenchymal cells, especially the bronchial epithelial cells. Compared with the control group, GRP78 positive staining in the lung tissues of the CS group was significantly increased, characterized by plenty of brown granules. Compared with the CS group, GRP78 positive staining in the lung tissues of NaHS + CS group was significantly decreased, and GRP78 positive staining in the lung tissues of PPG + CS group was increased. **(E)** P-IRE1 (red arrow) is localized to the cytoplasm of respiratory epithelial cells and mesenchymal cells. Compared with control group, p-IRE1 positive staining in the lung tissues of the CS group was significantly increased, characterized by a plenty of brown granules. Compared with CS group, p-IRE1 positive staining in the lung tissues of the NaHS + CS group was significantly decreased, and p-IRE1 positive staining in the lung tissues of the PPG + CS group was increased. Representative images (*n* = 4, respectively, in each group). Original magnification ×100 and ×400.

Immunohistochemistry showed that E-cadherin was decreased in the CS group and the PPG + CS group compared with the control group. The NaHS + CS group had more intense E-cadherin compared with the CS group. Vimentin and alpha-SMA are localized mainly in the cytoplasm of mesenchymal cells. Compared with the control group, alpha-SMA and Vimentin were more intense in the CS group and the PPG + CS group, especially in bronchial epithelial cells. The NaHS + CS group had decreased alpha-SMA and Vimentin compared with the CS group (Figure 3A).

### Endogenous H_2_S Inhibited CS-Induced Lung Epithelial Cell Endoplasmic Reticulum Stress in Rats

Western blot showed the ER stress markers p-IRE1, GRP94, ATF6 and sec-XBP1 were increased in the lung tissue of the CS group compared with the control group. Intraperitoneal injection of NaHS in the NaHS + CS group significantly decreased these markers compared with the CS group (Figures 3B,C). Meanwhile, intraperitoneal injection of PPG in the PPG + CS group increased these markers compared with the CS group (Figures 3B,C). Immunohistochemistry showed that GRP78 was expressed mainly in lung epithelial cells, and p-IRE1 was expressed in lung epithelial cells and smooth muscle cells. They were all strongly stained in the CS group and the PPG + CS group, and lightly stained in the control group and the NaHS + CS group (Figures 3D,E).

### H_2_S Inhibited Nicotine-Induced Endoplasmic Reticulum Stress in Human Bronchial Epithelial Cells *In vitro*

Because no standard methods for the preparation of cigarette smoke extract have been established and nicotine, as an important component of cigarette smoke extract, can directly induce EMT (Dasgupta et al., 2009; Pillai et al., 2015; Zhang et al., 2016; Du et al., 2018) and ER stress (Lin et al., 2017) in the lung, we employed a pure nicotine cell model for the mechanistic work. Nicotine concentration dependently increased the expression of ER stress markers and alpha-SMA, and nicotine also concentration dependently decreased E-cadherin in 16HBE cells (Supplementary Figure S2). And it has been reported in our previous study that nicotine also time-dependently increased the protein level of the ER stress marker (Lin et al., 2017). 40 μmol/L nicotine treated for 72 h significantly induced ER stress and EMT in 16HBE cells.

**FIGURE 4:**
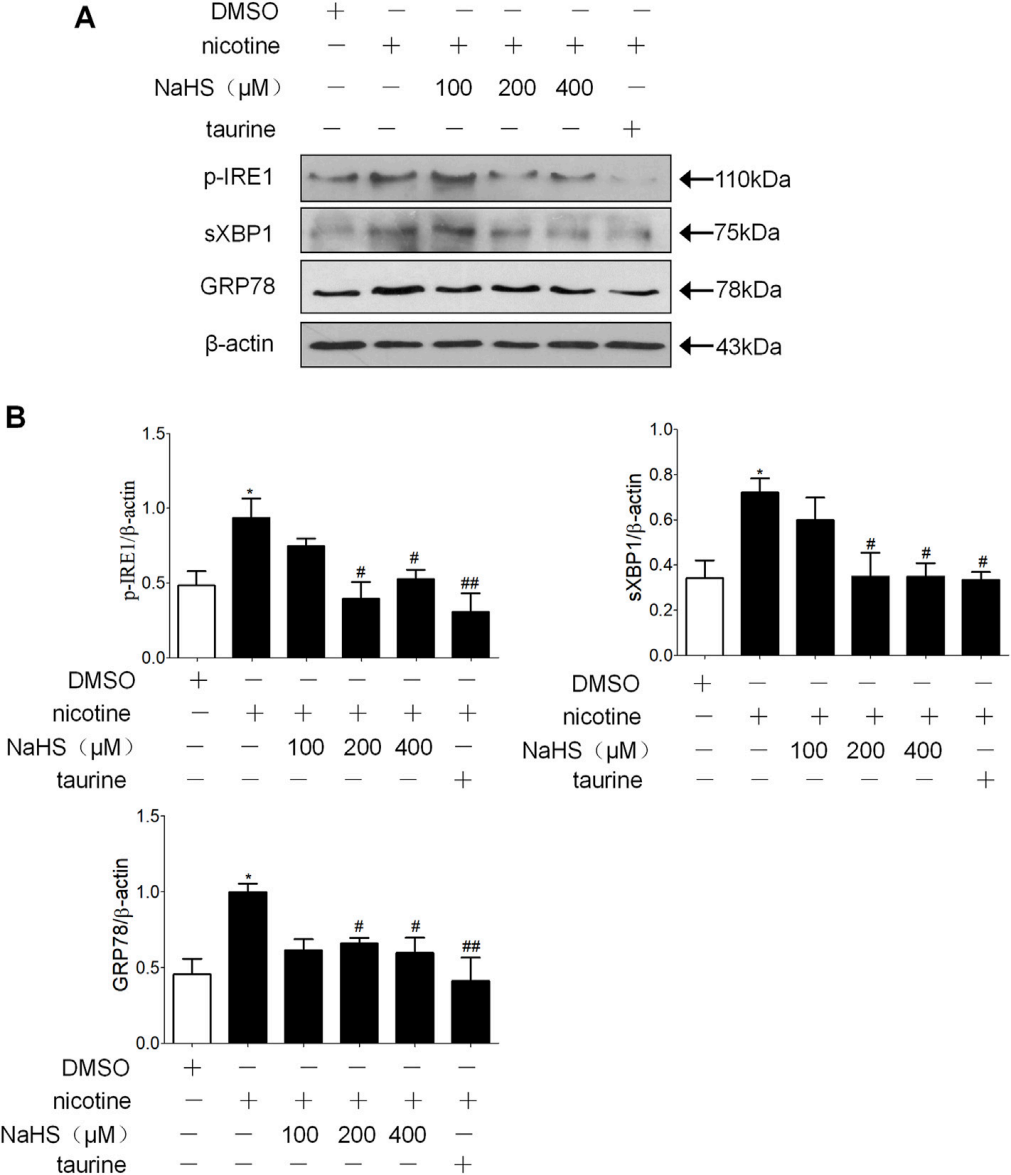
H_2_S and ER stress inhibitor taurine suppressed nicotine-induced ER stress in 16 HBE cells. **(A,B)** Nicotine (40 μmol/L) was used. Western blotting analysis of ER stress markers p-IRE1, sec-XBP1, and GRP78 protein expression in 16HBE cells, and relative intensity normalized to the expression of β-actin (*n* = 3, respectively, in each group). Values are expressed as mean ± SEM. *p < 0.05 vs. the DMSO group. ^**#**^ *p* < 0.05, ^**##**^ *p* < 0.01 vs. nicotine group.

Preincubation of 16HBE cells with NaHS and then treating them with nicotine resulted in a concentration-related inhibition of ER stress markers p-IRE1, sec-XBP1 and GRP78 formation compared with nicotine alone, appropriate concentration of NaHS (200 µM) reduced p-IRE1, sec-XBP1 and GRP78 formation to the greatest extent (Figures 4A,B).

### H_2_S Inhibited Nicotine-Induced Human Bronchial Epithelial Cell Epithelial Mesenchymal Transition by Regulating Endoplasmic Reticulum Stress *In vitro*

**FIGURE 5:**
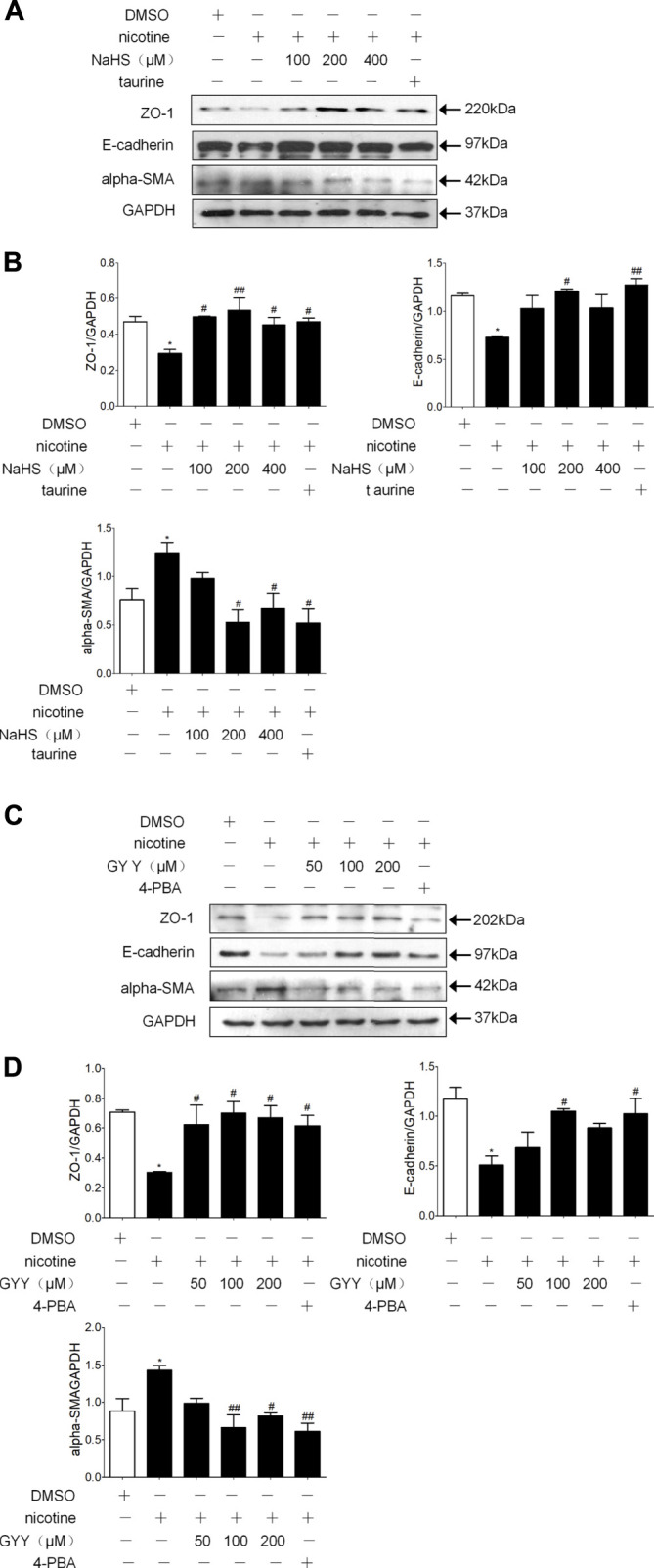
H_2_S and ER stress inhibitors taurine and 4-PBA suppressed nicotine-induced changesin the expression of EMT markers in 16HBE cells. **(A,B)** Western blotting analysis of ZO-1, E-cadherin, and alpha-SMA protein expression in 16 HBE cells, and relative intensity normalized to the expression of GAPDH (*n* = 3, respectively, in each group). 200 μmol/L NaHS reversed nicotine induced-EMT to the greatest extent. Values are expressed as mean ± SEM. *p < 0.05 vs. the DMSO group. ^**#**^ *p* < 0.05, ^**##**^ *p* < 0.01 vs. nicotine group. **(C,D)** Western blotting analysis of ZO-1, E-cadherin, and alpha-SMA protein expression in 16HBE cells, and relative intensity normalized to the expression of GAPDH (*n* = 3, respectively, in each group). 100 μmol/L GYY4137 reversed nicotine induced-EMT to the greatest extent. Values are expressed as mean ± SEM. *p < 0.05 vs. the DMSO group. ^**#**^ *p* < 0.05, ^**##**^ *p* < 0.01 vs. nicotine group.

Preincubation of 16 HBE cells with H_2_S donor NaHS or GYY4137 and then treating with nicotine resulted in a concentration-related down regulation of alpha-SMA and up regulation of ZO-1 and E-cadherin compared with nicotine alone. An appropriate concentration of NaHS (200 µM) reduced the expression of alpha-SMA and increased the expression of ZO-1 and E-cadherin to the greatest extent (Figures 5A,B). Appropriate concentration of GYY4137 (100 µM) reduced the expression of alpha-SMA and increased and the expression of E-cadherin to the greatest extent (Figures 5C,D). Pre-exposure 16HBE cells to ER stress inhibitor taurine or 4PBA all significantly reduced alpha-SMA expression and increased ZO-1 and E-cadherin expression (Figure 5).

### H_2_S Inhibited Nicotine-Induced Enhancement of the Migration Ability of Human Bronchial Epithelial Cells by Regulating ER Stress *In vitro*

**FIGURE 6:**
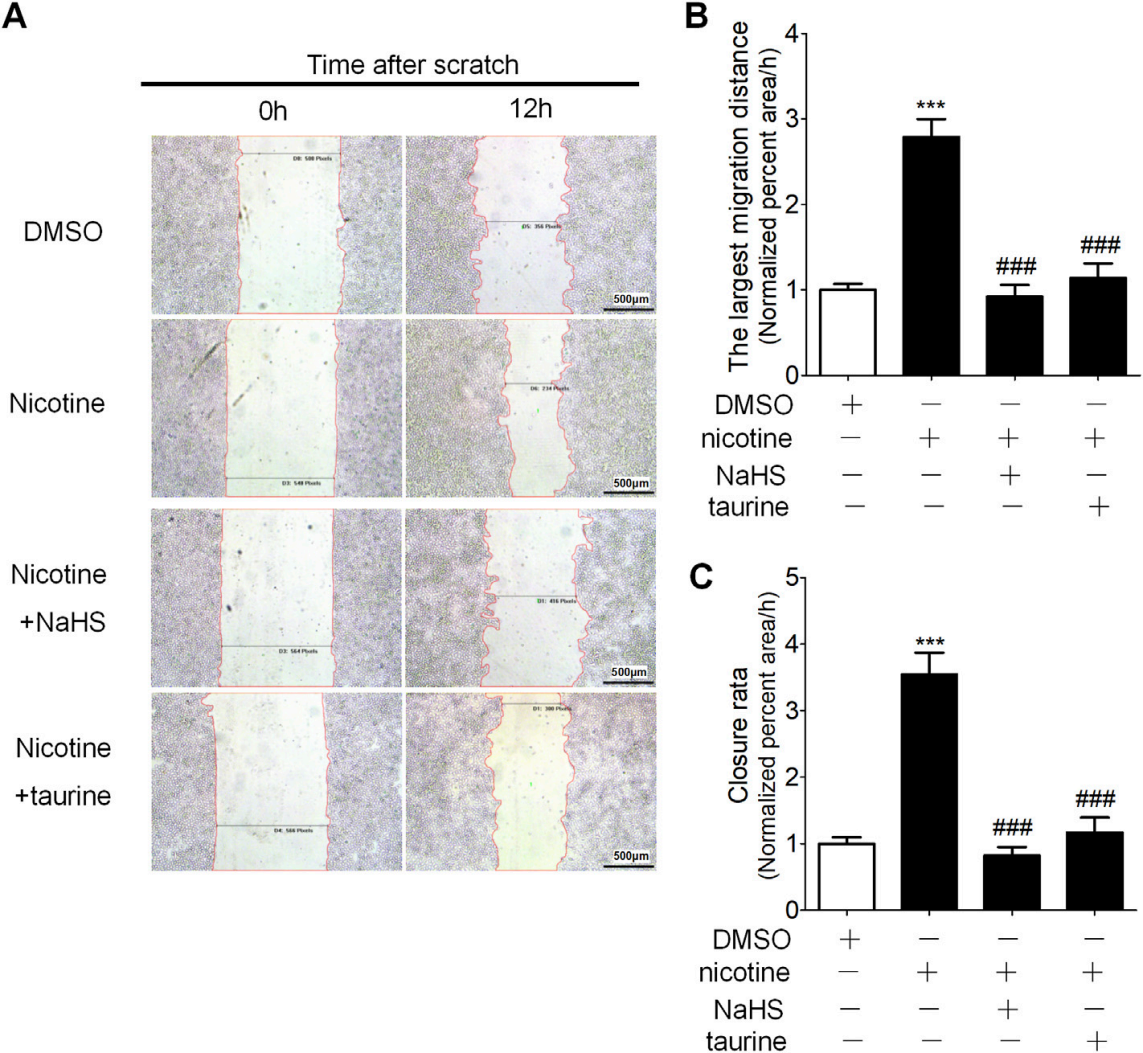
NaHS and ER stress inhibitor taurine reduced nicotine-induced enhancement of cell migration ability in 16HBE cells. **(A)** Wound healing assay on the migration of 16 HBE cells. The percentage of the wound healing was calculated as the largest migration distance (the shortest width of wound at 0 h—the shortest width of the wound at 12 h/the width of the wound at 0 h) and the closure rate (the area of the wound at 0 h—the area of wound at 12 h/the area of the wound at 0 h). **(B,C)** The largest migration distance and the closure rate calculated by IPP software after wound healing assay. Results are presented from 6 independent experiments (*n* = 6, respectively, in each group). Values are expressed as mean ± SEM. ***p < 0.001 vs. the DMSO group. ^**###**^ *p* < 0.001 vs. nicotine group.

Wound healing assay showed nicotine stimulation promoted cell migration of 16HBE cells, and preincubation of 16HBE cells with taurine or NaHS significantly retarded cell migration compared with nicotine alone (Figure 6).

### H_2_S Inhibited Human Bronchial Epithelial Cell Epithelial Mesenchymal Transition *via* Suppressing IRE1 Signal Pathway and the Activation of Smad2/3

**FIGURE 7:**
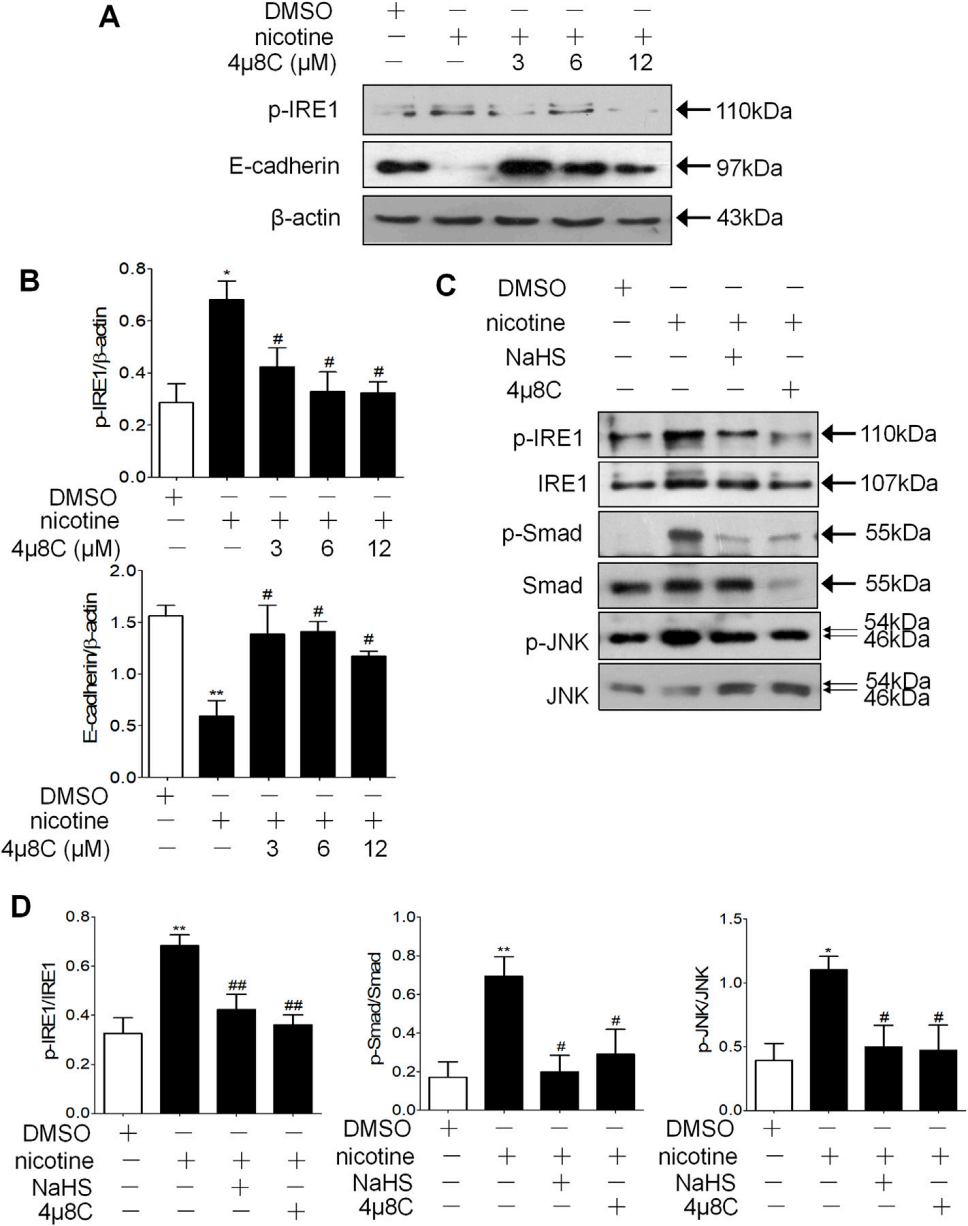
NaHS and IRE1 activation inhibitor 4μ8C inhibited the phosphorylation of IRE1 and Smad2/3 to inhibit EMT in 16HBE cells. **(A,B)** Western blotting analysis of p-IRE1 and E-cadherin protein expression in 16HBE cells, and relative intensity normalized to the expression of β-actin (*n* = 3, respectively, in each group). Values are expressed as mean ± SEM. *p < 0.05 vs. the DMSO group and ^**#**^ *p* < 0.05 vs. nicotine group. **(C,D)** Western blotting analysis of the ratio of p-IRE1/IRE1, p-Smad2/3/Smad2/3 and p-JNK/JNK in 16HBE cells. Results are presented from 3 independent experiments (*n* = 3, respectively, in each group). Values are expressed as mean ± SEM. *p < 0.05, **p < 0.01 vs. the DMSO group. ^**#**^ *p* < 0.05, ^**##**^ *p* < 0.01 vs. nicotine group.

IRE1 activation inhibitor 4μ8C decreased the phosphorylation of IRE1 and increased the protein expression level of E-cadherin compared with nicotine alone in a concentration-dependent manner (Figures 7A,B). The 200 μM NaHS or 6 μM 4μ8C preincubation of the 16HBE cells significantly reduced the formation of p-JNK and p-Smad2/3 (Figures 7C,D). Prolonged ER stress increases the phosphorylation of IRE1, which increases the phosphorylation of JNK, in our research, 6 μM 4μ8C or 200 μM NaHS inhibited nicotine-induced the activation of IRE1-JNK pathway and Smad2/3.

## Discussion

The peripheral airways are the most vulnerable areas of the respiratory tract to CS, pollutants, and toxic substances. Therefore, inflammation, injury, and differentiation pathological changes are more common in the peripheral airways (Higham et al., 2019). Recently, a large-scale epidemiological research of 50,479 adults in China reported that the small-airways dysfunction associated to the presence of COPD (Xiao et al., 2020)**.** Small-airways, dysfunction preceded both the detection of emphysema by imaging methods and spirometric evidence of COPD (Hogg et al., 2004). CS induced EMT in lung epithelial cells can contribute to COPD remodeling events (Sohal et al., 2010; Liu et al., 2010; Sohal et al., 2011; Veljkovic et al., 2011; Eurlings et al., 2014). The results that were found in this research showed EMT occurred in non-COPD smokers’ and COPD patients’ lung epithelial cells, especially in the bronchial epithelial cells (Figure 1). We got a similar result to the previous studies (Sohal et al., 2010) that showed EMT was indeed active in smokers’ airways, in particular in the airways of smokers with COPD. EMT markers significantly correlated with pulmonary function in COPD patients (Takizawa et al., 2001; Sohal et al., 2010). EMT markers significantly elevated in non-COPD smokers implies that EMT may be involved in the occurrence of COPD. But it still needs further work to investigate the specific connection, especially the signal pathway, between EMT and COPD, and there is a lack of an effective drug to reverse EMT and the occurrence and development of COPD.

H_2_S has been confirmed to play a protective role in tissue damage in the lungs, heart, brain, gastrointestinal tract, liver, and other organs caused by a variety of stressors. According to our previous research*,* the level of H_2_S in serum was significantly higher in non-smokers compared with smokers, and it was significantly higher in smokers with stable COPD and healthy smokers than in smoking patients who suffer from acute exacerbations of COPD (Chen et al., 2005). We also identified that H_2_S significantly alleviated CS-induced lung pathological damage and lung function damage. Exogenous H_2_S reduced the pathology scores of lung tissue and was antioxidant and anti-inflammatory (Chen et al., 2011). The results that were found in this research showed exogenous administration of NaHS in a COPD rat model established by passive smoking exposure significantly reversed the pathological damage in lung tissue and small airway fibrosis, whereas exogenous administration of PPG aggravated lung tissue damage and small airway fibrosis, so the endogenous H_2_S played an inhibitory effect on small airway fibrosis (Figure 2).

Inhibition of endogenous H_2_S by CSE inhibitor PPG resulted in EMT-like features, increased the protein expression of vimentin and decreased the protein expression of E-cadherin. Exogenous administration of H_2_S decreased these effects in the non-small cell lung cancer cell line A549 stimulated by TGF-β1 (Fang et al., 2010). In our previous research, H_2_S inhibited EMT occurrence in human bronchial epithelial cell line 16HBE induced by TGF-β1 (Liao et al., 2015). The results that were found in this research showed exogenous administration of NaHS in the COPD rat model established by passive smoking exposure significantly reversed EMT, whereas intraperitoneal injection of PPG aggravated EMT in lung epithelial cells (Figure 3). This is consistent with the trend of small airway fibrosis in these groups. These results suggest that in lung epithelial cells, endogenous H_2_S may have an inhibitory effect on EMT related small airway fibrosis.

It has been confirmed that the main mutagenicity components of CS condensates exist in alkaline and slightly acidic parts, while the mutagenicity was not detected in neutral parts containing polycyclic aromatic hydrocarbons (Kier et al., 1974; Hutton and Hackney, 1975). It suggests that mutagenic components in CS condensates mainly exist in the water-soluble part, and the water-soluble part of CS is more easily dissolved and absorbed by the mucus on the alveolar surface (Ji and Chen, 1996). Acrolein, nicotine, and acetylaldehyde are among the most important and relevant chemicals in CS (Comer et al., 2014). Acrolein is highly toxic, a large number of studies have shown that acrolein can induce apoptosis (Kern and Kehrer, 2002; Roy et al., 2010), but few studies have shown that it can induce EMT and ER stress. When tobacco smoke reaches the small airways and alveolar surface, the nicotine is rapidly absorbed. On average, about 1–0.5 mg of nicotine is absorbed systemically during smoking on average (Benowitz and Iii, 1984). Nicotine is directly associated with COPD. Evaluation of electronic nicotine delivery systems in different models has demonstrated involvement in pathways related to chronic pulmonary diseases (Canistro et al., 2017; Singh et al., 2019; Mikheev et al., 2020). Inhaled nicotine induced bronchial epithelial cell senescence and apoptosis *via* ROS-mediated autophagy impairment in COPD patients (Bodas et al., 2016). Nicotine can promote EMT in the lungs. Maternal nicotine exposure induces EMT in rat offspring lungs (Chen et al., 2015). Nicotine can increase malignancy through EMT in lung cancer (Dasgupta et al., 2009; Pillai et al., 2015; Zhang et al., 2016; Du et al., 2018). Nicotine can directly induce ER stress response (Pelissier-Rota et al., 2015; Barra et al., 2017; Gonzales et al., 2021; Jiang et al., 2021). In our previous study, we used a nicotine exposure model to investigate whether H_2_S can inhibit cigarette smoke-induced ERS and apoptosis in bronchial epithelial cells (Lin et al., 2017). Our previous study confirmed that nicotine concentration and time-dependently increased the expression of ER stress associated apoptosis marker in human bronchial epithelial cells, participating in the progression of COPD (Lin et al., 2017).

ER stress can activate classic Smad, Wnt/β-catenin and Src protein kinase families, thus inducing EMT in alveolar epithelial cells (Tanjore et al., 2011; Zhong et al., 2011; Zhang et al., 2012) ER stress participates in EMT and oncogenesis (Sheshadri et al., 2014). The results that were found in this research showed H_2_S inhibited lung epithelial cell ER stress and EMT in a COPD rat model established by passive smoking exposure (Figure 3). In the 16HBE cells exposed to nicotine, a suitable concentration of GYY4137 or NaHS not only inhibited nicotine-induced ER stress, but also inhibited nicotine-induced enhancement of cell migration ability and EMT (Figure 5). ER stress nonspecific inhibitor taurine or 4-PBA also suppressed the enhancement of cell migration ability and EMT in 16HBE cells exposed to nicotine (Figure 6). A suitable concentration of H_2_S can simulate this effect. The results shown above indicate that H_2_S can inhibit EMT through regulating ER stress.

We also investigated the signaling pathways of ER stress to influence the occurrence of EMT in bronchial epithelial cells by H_2_S. We found that the inhibition of IRE1 signaling pathways significantly alleviated the nicotine-induced down-regulated expression of E-cadherin (Figure 7). This finding is supported by a previous report of alveolar epithelial cells (Zhong et al., 2011), which suggests that inhibition of IRE1 signaling pathways can reverse EMT. Furthermore, we identified that both 4μ8C and H_2_S suppressed the activation of IRE1, Smad2/3, and JNK (Figure 7). Excessive ER stress results in kinase activity of IRE1 along with phosphorylation of JNK. In our research, H_2_S and 4μ8C inhibited nicotine-induced IRE1 kinase activation, thus inhibiting the activation of IRE1 downstream signal molecules including JNK and Smad2/3. Our finding is supported by previous reports of alveolar epithelial cells (Zhong et al., 2011), which suggest that p-IRE1 and EMT-related signal molecule Smad2/3 play an essential role in EMT mediated by ER stress, and pretreatment of H_2_S suppressed the phosphorylation of Smad2/3 stimulated by TGF-β1. Both Smad2/3 inhibitor and H_2_S could inhibit ER stress-induced EMT (41). In this research, we identified that H_2_S could inhibit bronchial epithelial cell EMT through suppressing the activation of IRE1 and its downstream signal molecule Smad2/3. Our finding suggested that H_2_S could also inhibit ATF6 activation. Another report reported that activation of PERK and ATF can cause EMT (Sheshadri et al., 2014). Therefore, it is not clear that H_2_S can directly act on IRE1 or regulate the upstream signal pathway of ER stress. According to recent reports, H_2_S can regulate ER stress in different organ systems through different action sites. H_2_S can directly persulfidate protein kinase and regulate the protein kinase activity (Du et al., 2021). H_2_S may reduce ER stress via regulation of Ca^2+^ channel sulfhydration (Hennig and Diener, 2009; Luciani et al., 2009; Moccia et al., 2011). H_2_S may regulate ER stress through its antioxidant effect. Some studies have revealed that H_2_S can both regulate oxidative stress and ER stress (Hu et al., 2017; Majumder et al., 2018; Yi et al., 2018). In addition, H_2_S can inhibit homocysteine-induced neurocyte ER stress and apoptosis by up-regulation of the brain-derived neurotrophic factor (BDNF)-TrkB pathway (Wei et al., 2014). Our research identified that H_2_S significantly inhibiting CS-induced ER stress, thus inhibited ER stress-mediated EMT.

There are several limitations to our investigation. Firstly, we have not figured out the specific signal pathway of H_2_S on ER stress. Knock out the gene in the IRE1 signal pathway or Smad2/3 may be a better way to figure out the importance of these signal molecules in ER stress mediated EMT. Secondly, the number of lung tissue samples was too small. We are ready to recruit more patients, including non-smoking COPD patients and COPD patients in different GOLD stages, in the future study. Immunofluorescence staining and co-staining with EMT marker and ER stress marker is a better way than immunohistochemical staining to identify the cells that undergo EMT and ER stress. Further studies are needed to overcome these shortcomings.

In conclusion, it is proved for the first time that H_2_S can inhibit CS- or nicotine-induced ER stress and EMT in bronchial epithelial cells. The IRE1 signal pathway and its downstream signal molecule p-Smad2/3 may be responsible for the inhibitory effect of H_2_S. These findings suggest a possible protective role of anti-fibrosis for H_2_S in the pathogenesis of COPD.

## Data Availability

The raw data supporting the conclusion of this article will be made available by the authors, without undue reservation.

## Abbreviations

ATF6, activating transcription factor 6; alpha-SMA, alpha-smooth muscle actin; BDNF, brain-derived neurotrophic factor; CS, cigarette smoke; COPD, chronic obstructive pulmonary disease; CSE, cystathionine-γ-lyase; CBS, cystathionine-β-synthase; CHOP, C/EBP homologous protein; EMT, epithelial mesenchymal transition; ER, endoplasmic reticulum; FEV1, forced expiratory volume in the first second; FVC, forced vital capacity; FBS, fetal bovine serum; GOLD, Global initiative for chronic Obstructive Lung Disease criteria; GRP78, glucose-regulated protein 78; GYY4137, morpholin-4-ium-4-methoxyphenyl-(morpholino)-phosphinodithioate; H_2_S, hydrogen sulfide; HE, hematoxylin and eosin; IRE1, inositol requiring enzyme 1; InsP3Rs, inositol-1,4,5-triphosphate receptor; JNK, c-Jun N-terminal kinase; MST, mercaptopyruvate sulfurtransferase; PPG, propargylglycine; 4PBA, 4-phenyl butyric acid; PERK, PKR-like ER Kinase; RyRs, ryanodine receptors; SERCA, sarco/endoplasmic reticulum Ca^2+^-ATPase; SIRT1, silent mating type information regulation 2 homolog 1; TGF-β1, transforming growth factor β1; XBP1, x-box binding protein1; ZO-1, zonula occludens-1.
