# Supplementary material for: Hydrogen Sulfide Inhibits Bronchial Epithelial Cell Epithelial Mesenchymal Transition Through Regulating Endoplasm Reticulum Stress
Source: Front Mol Biosci. 2022 Apr 12;9:828766. doi: 10.3389/fmolb.2022.828766 (PMC9039047; doi:10.3389/fmolb.2022.828766)
Supplement: Supplementary file 1 [file DataSheet1.doc]

**Supplementary Figure 1.** Nicotine concentration dependently increased the expression of ER stress markers and changed the expression of EMT markers in 16HBE cells. **(A, B)** Western blotting analysis of ER stress markers p-IRE1 and sXBP1 protein expression in 16HBE cells, and relative intensity normalized to the expression of GAPDH (n = 3, respectively, in each group). With the increase of nicotine concentration, p-IRE1 and sXBP1 protein expression increased gradually. Compared with the control group, the expression of p-IRE1 and sXBP1 in the 40μmol/L nicotine group significantly increased by 90.54% (*P* < 0.01) and 467.57% (*P <* 0.05). **(C, D)** Western blotting analysis of a-SMA and E-cadherin protein expression in 16HBE cells, and relative intensity normalized to the expression of β-actin (n = 3, respectively, in each group). With the increase of nicotine concentration, α-SMA protein expression increased and E-cadherin protein expression decreased gradually. Compared with the control group, the expression of a-SMA in the 40μmol/L nicotine group significantly increased by 304.32% (*P* < 0.05) and the expression of E-cadherin in the 40μmol/L nicotine group significantly decreased by 52.25% (*P* < 0.05). Values are expressed as mean ± SEM. * *P* < 0.05, ** *P* < 0.01 vs. 0μmol/L nicotine group.

**Supplementary Figure 2.** Changes in the protein level of CSE in the lung homogenates of rats. **(A, B)** Western blotting analysis of CSE protein expression in the lung homogenates of rats, and relative intensity normalized to the expression of β-actin. Results are presented for n = 8 mice per group and of 3 independent experiments (n = 3). Values are expressed as mean ± SEM. ** P < 0.01 vs. control group and ## P < 0.01 vs. CS group.
