# Supplementary figures and images for: Hydrogen Sulfide Inhibits Bronchial Epithelial Cell Epithelial Mesenchymal Transition Through Regulating Endoplasm Reticulum Stress
Source: Front Mol Biosci. 2022 Apr 12;9:828766. doi: 10.3389/fmolb.2022.828766 (PMC9039047; doi:10.3389/fmolb.2022.828766)

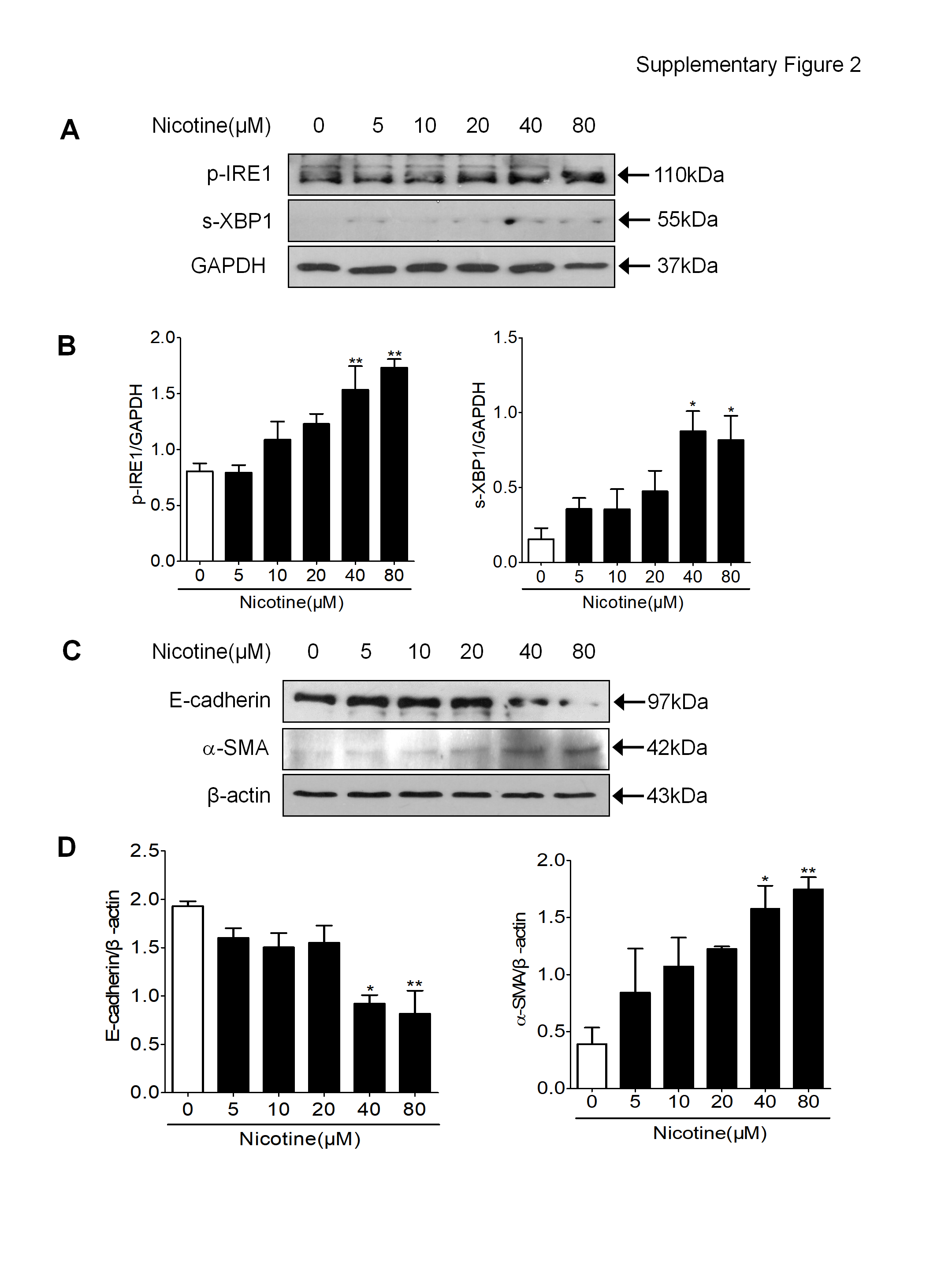

Supplement: Supplementary file 2 [file Image2.TIF]

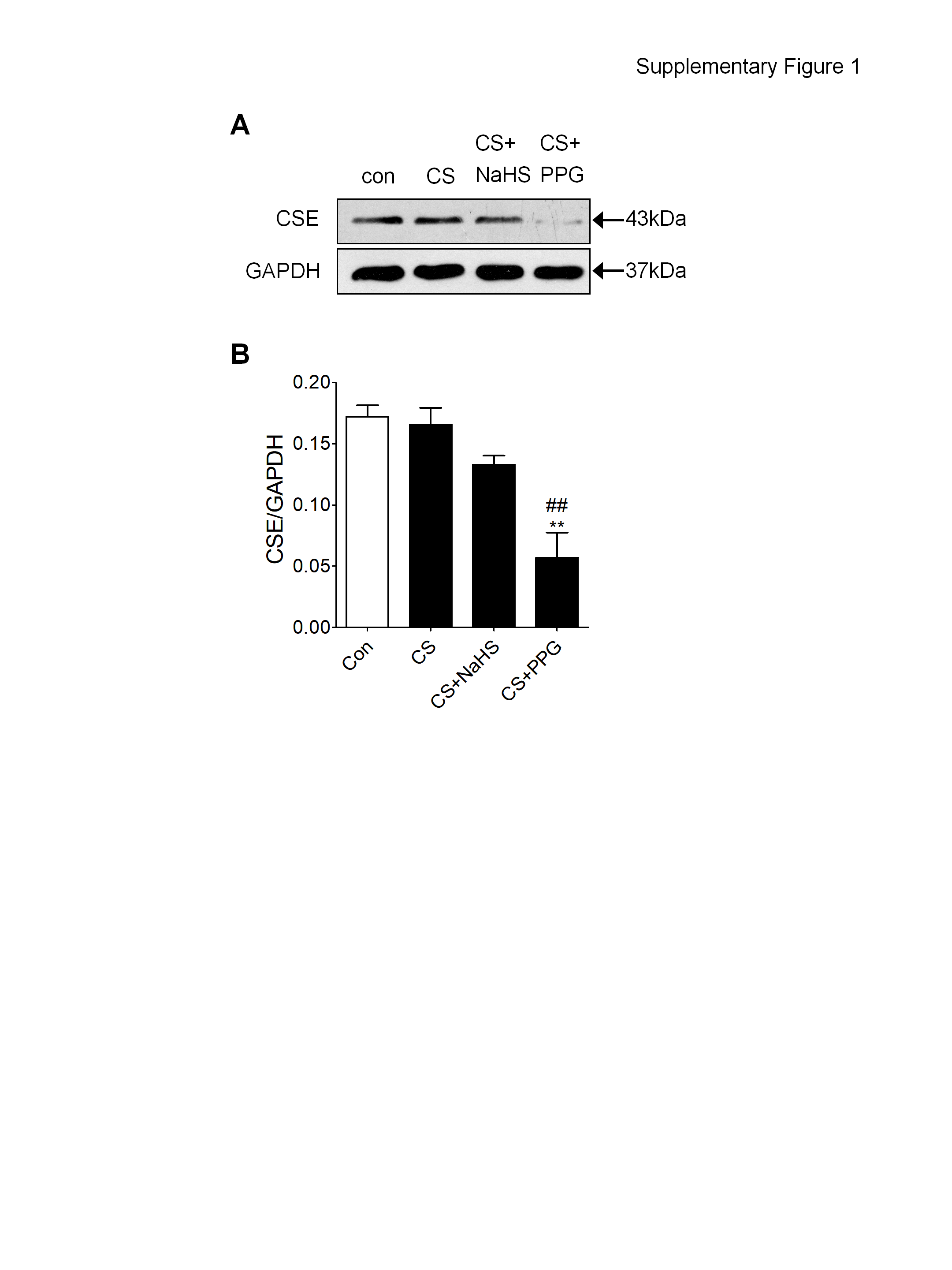

Supplement: Supplementary file 3 [file Image1.TIF]
